# CT scan screening is associated with increased distress among subjects of the APExS

**DOI:** 10.1186/1471-2458-10-647

**Published:** 2010-10-26

**Authors:** Christophe Paris, Marion Maurel, Amandine Luc, Audrey Stoufflet, Jean-Claude Pairon, Marc Letourneux

**Affiliations:** 1 Nancy University Hospital, 54000 Nancy, France; 2 INSERM 954, 54505 Vandoeuvre-les-Nancy Cedex - France; 3 EA 2304, 93526 Saint-Denis Cedex, France; 4 INSERM, Unité 955, F-94010 Creteil, France; 5 CHI Creteil, F-94010 Creteil, France; 6 University Hospital. Occupational diseases Dept. 14031 Caen Cedex, France

## Abstract

**Background:**

The aim of this study was to assess the psychological consequences of HRCT scan screening in retired asbestos-exposed workers.

**Methods:**

A HRCT-scan screening program for asbestos-related diseases was carried out in four regions of France. At baseline (T1), subjects filled in self-administered occupational questionnaires. In two of the regions, subjects also received a validated psychological scale, namely the psychological consequences questionnaire (PCQ). The physician was required to provide the subject with the results of the HRCT scan at a final visit. A second assessment of psychological consequences was performed 6 months after the HRCT-scan examination (T2). PCQ scores were compared quantitatively (t-test, general linear model) and qualitatively (chi²-test, logistic regression) to screening results. Multivariate analyses were adjusted for gender, age, smoking, asbestos exposure and counseling.

**Results:**

Among the 832 subjects included in this psychological impact study, HRCT-scan screening was associated with a significant increase of the psychological score 6 months after the examination relative to baseline values (8.31 to 10.08, p < 0.0001, t-test). This increase concerned patients with an abnormal HRCT-scan result, regardless of the abnormalities, but also patients with normal HRCT-scans after adjustment for age, gender, smoking status, asbestos exposure and counseling visit. The greatest increase was observed for pleural plaques (+3.60; 95%CI [+2.15;+5.06]), which are benign lesions. Detection of isolated pulmonary nodules was also associated with a less marked but nevertheless significant increase of distress (+1.88; 95%CI [+0.34;+3.42]). However, analyses based on logistic regressions only showed a close to significant increase of the proportion of subjects with abnormal PCQ scores at T2 for patients with asbestosis (OR = 1.92; 95%CI [0.97-3.81]) or with two or more diseases (OR = 2.04; 95%CI [0.95-4.37]).

**Conclusion:**

This study suggests that HRCT-scan screening may be associated with increased distress in asbestos-exposed subjects. If confirmed, these results may have consequences for HRCT-scan screening recommendations.

## Background

Asbestos is responsible for both non-malignant diseases such as pleural plaques and asbestosis and malignant diseases such as mesothelioma and lung cancer [1]. Mesothelioma is known to occur even at low levels of asbestos exposure. Recent publications have also reported that lung cancer may occur in patients exposed to asbestos at lower levels than previously demonstrated [2]. Thoracic High Resolution Computed Tomography (HRCT) has been clearly demonstrated to be more sensitive and specific than Chest X-Rays for the diagnosis of asbestos-related diseases even at early stages [3] and for lung cancer [4]. The question of CT scan screening for lung cancer is currently debated in both high-risk populations exposed to tobacco smoke or occupational carcinogens, as no definite proof of the benefit of such screening has been published [5]. Recommendations for potential applications of CT scan in lung cancer screening among asbestos-exposed subjects have been published [6] and several lung cancer screening programs have subsequently been reported [7,8]

To date, there is no evidence that any intervention can modify the natural history of any asbestos-related condition, and therefore, that the necessary pre-conditions for any screening programme have not been yet met. On the other hand, French regulations lead to several advantages to former workers suffering of an asbestos-related disease such as compensations and the right to an earlier retirement, even for pleural plaques. As a result regarding these social advantages and despite the absence of a medical benefit, screening of asbestos-related diseases is recommended in France under some specific criteria.

In this context, the impact of adverse effects such as negative psychological impact in large screened populations may be important, particularly in view of the high prevalence of false-positive results (namely benign isolated pulmonary nodules requiring periodic survey) [9]. However, to date only a few data have been published on the psychological consequences of CT scan lung cancer screening programs [10-12], reporting only minimal distress at 6 months in ever smokers in relation to screening. To the best of our knowledge, only one study [13] has evaluated the possible psychological impact of CT screening in asbestos-exposed subjects. This study found no significant psychological differences one year after CT scan between subjects who received clear results at inclusion and those who were submitted to additional examinations because of positive findings, in a sample of 601 subjects surveyed for asbestos-related diseases. Only sparse documentation is available regarding the specific distress related to asbestos exposure. Most of these previous studies reported the absence of any observed psychological distress in subjects exposed to asbestos [14,15]. Evaluation of the psychological impact of CT-scan screening was one of the objectives of the French multiregional asbestos-post exposure survey ordered by national authorities. We recently published a study showing that asbestos-exposed subjects exhibited significantly higher levels of negative psychological impact compared to a control group at baseline of a screening program [16].

We therefore hypothesized that the psychological impact related to screening may be more pronounced in this particular population. The aim of this study was to assess the long-term psychological consequences of a CT scan screening in the population of the asbestos post-exposure survey (APExS).

## Methods

The overall design of this study has been previously published [16,17].

### Global design

A large-scale screening program for asbestos-related diseases (the Asbestos Post-Exposure Survey APEXS) was carried out in four regions of France between October 2003 and December 2005. The target group for the screening program comprised unemployed or retired asbestos-exposed workers covered financially by French National Health Insurance. Recruitment procedures were based on television, newspapers, mailing or systematic invitations at national health insurance centers in each region. All volunteer subjects were asked to complete a standardized questionnaire describing all jobs held throughout the subject's working life, as well as specific asbestos-exposing tasks. Questionnaires were analyzed by industrial hygienists (IH) or trained national health insurance agents depending on areas. The level of exposure was defined for each subject's entire career and classified into four classes: high (defined as "continuous exposure for at least one year" or "discontinuous exposure for at least 10 years"), low (passive exposure), moderate (all other occupational exposure) and nil (no exposure). Only subjects with a reliable occupational asbestos exposure as assessed by IH, regardless of the level and occupational characteristics, and with no known occupational asbestos-related disease were invited to undergo a free screening program comprising clinical examination and pulmonary function tests with the chest physician of their choice, and chest X-rays and spiral CT-scan performed by program-approved radiologists. A total of 20,157 subjects volunteered to participate in the APEXS and 16,885 subjects returned the completed occupational questionnaire; 13,859 (82.1%) of these subjects were eligible for the screening program in terms of their National Health Insurance cover and more than half of them (7,275; 52.5%) underwent chest HRCT. Subjects whose HRCT reports were not sent to the coordination center (n = 734) or who presented incomplete data (n = 709), as well as non-exposed subjects (n = 32), were excluded from further analysis.

Recommended HRCT acquisition parameters were defined in accordance with the French Thoracic Imaging Society guidelines. Radiologists who participated in the program received guidelines on how to perform HRCT for the diagnosis of asbestos-related benign diseases as well as specific training in the interpretation of HRCT from experienced radiologists and occupational physicians. Recommendations were given for the survey of isolated pulmonary nodules, according to the Fleishner Society [18].

For the purposes of this study, the presence or absence of radiological abnormalities (asbestos-related diseases, pulmonary nodules with a diameter ≥ 5 mm, other disorders) was rated only on the basis of HRCT reports by radiologists blinded to clinical data. More precise definitions are given elsewhere [17]. HRCT scan results and counseling had to be given to the patient by the general practitioner or the respiratory physician during a final visit.

### Study design

The present study, designed to investigate the psychological impact of CT-scan screening, is an ancillary study of the national screening program. For this purpose, subjects living in two of the four regions of the study also received at baseline (defined as T1) together with the occupational questionnaire, a specific questionnaire to assess risk factors associated with asbestos exposure distress. Subjects having sent both questionnaires but with not known asbestos exposure were used as a control group for distress assessment. For the purposes of this study, all patients consecutively enrolled between December 2004 and December 2005 (n = 1184) with a first recorded PCQ and an identified HRCT scan examination were invited to fill in the PCQ again, 6 months after the date of the HRCT scan (defined as T2). Among the 867 returns (73.2%), only 832 subjects completely filled in all the questionnaires.

Distress was measured using the Psychological Consequences Questionnaire (PCQ). Initially developed by Cockburn et al. [19]to assess the psychological consequences of breast cancer screening by mammography, the PCQ is a 12-item self-report instrument measuring the effect of screening on the individual's functioning on emotional, physical and social life domains. A French version of this scale has been validated by Maziade et al.[20]. The PCQ was adapted for use in the present study with slight modifications in its published form, as questions were asked in relation to asbestos-related diseases in general (see online additional file 1). The response options ranged from 0 (not at all) to 3 (quite a lot of the time) and a score was calculated by summing each response for each subscale and for the global scale. Each study participant was asked to complete general characteristics (age, gender, smoking status), self-perception of current and future health status and their opinions and knowledge about asbestos-related diseases. Self-assessment by the subjects of the intensity asbestos exposure was also used in the study.

The project was approved by the Cochin Hospital ethics committee in Paris. All patients received information on the study and gave their written informed consent to the radiologist for the increased radiation dose delivered by HRCT.

### Statistical analysis

Only subjects for whom the two questionnaires were available were included in the present analyses. Analyses of the PCQ score used quantitative definitions (PCQ values at T1, T2 and T2-T1 difference values) as well as a qualitative approach. In order to obtain a reference value for each gender, an abnormal global score was defined as a value greater than the 95^th ^percentile of the distribution of the PCQ scores calculated separately in males (n = 210) and females (n = 226) with no asbestos exposure in their own opinion as assessed by the initial questionnaire. These subjects were derived from the initial sample of volunteers from the same two regions. This conservative calculation is independent of the distribution function of the score. Subjects were then classified into two categories (normal/abnormal) according to their PCQ global score as previously published [16].

Analyses were conducted in order to describe PCQ scores at baseline and during follow-up according to the results of HRCT scan screening. Both univariate (t-test, paired t-test, Chi-square and McNemar tests, as relevant) and multivariate analyses (general linear model - GLM -, logistic regression - LR -) were performed. In every case, multiple models were adjusted for age, gender, smoking status, self-assessment of asbestos exposure and counseling. Power calculation showed a statistical power of 99% for a 25% increase of the PCQ score, but only 25% for an OR of 2.0, according to the number of subjects with abnormal HRCT results. Data were analyzed by SAS software (SAS Institute, release 9.2, USA).

## Results

The study population comprised 92.3% males with a mean age of 62 years; 9.1% of the subjects were smokers (table 1). HRCT scan results were not available for 160 of the 832 patients (reports or CD-ROM not available in the centers) and no abnormalities were found in another 213 patients. The remaining 459 patients (68.3% of patients with a known HRCT scan result) presented at least one lesion. Isolated pleural plaques were present in 113 patients (16.8%), isolated interstitial abnormalities compatible with asbestosis were present in 67 subjects (11.3%) and isolated pulmonary nodules were present in 79 subjects (11.8%). Other abnormalities mainly consisted of emphysema, bronchial abnormalities, calcified nodules or nonspecific sequelae.

**Table 1:** Description of the population (n = 832)

Variables	N (%)
**Gender**	
Male	768 (92.3)
Female	64 (7.7)
	
**Age**	
**< 60 years**	328 (39.4)
**60-74 years**	455 (54.7)
**≥ 75 years**	49 (5.9)
Mean (SD) [Range]	62.27 (7.78) [36.0-85.0]
	
**Smoking status**	
Non-smoker	381 (45.8)
Former smoker	375 (45.1)
Smoker	76 (9.1)
	
**Self-assessment of asbestos exposure**	
Nil/Light	94 (11.3)
Moderate	333 (40.0)
Heavy	216 (26.0)
Do not know	189 (22.7)
Counseling visit	
No	268 (32.2)
yes	564 (67.8)

**CT scan results**	
Normal	213 (25.6)
Abnormal	459 (55.2)
*Isolated pulmonary nodules (only)*	*79 (9.5)*
*Pleural plaques (only)*	*113 (13.6)*
*Asbestosis (with or without pleural plaques)*	*67 (8.0)*
*Other diseases (only)*	*151 (18.1)*
*Two or more of the above diseases*	*49 (5.6)*
Unknown results	160 (19.2)

Results are expressed as means and SD (age) or as numbers and percent (other variables) as relevant

According to our definition of abnormal PCQ score, 32.6% of subjects demonstrated an abnormal PCQ score at T2 compared to 20.5% before screening (p < 0.001) (Table 2). Moreover, 19.0% of the 661 subjects in whom the PCQ score was considered to be normal at baseline presented an abnormal PCQ score 6 months later (p < 0.0001, McNemar test). At baseline, only self-assessment of exposure was significantly associated with the PCQ score. At T2, an association was observed with this variable, but also with smoking status. Patients who attended a counseling visit had a significantly higher score at baseline but not at T2. A significant increase of PCQ score was observed for all categories of variables except in females. The proportion of patients with an abnormal PCQ score according to HRCT scan results was higher than at T1, ranging from 26.8% (normal HRCT scan) to 42.9% (two or more lesions) (Table 3). The most marked increases in the proportion of patients with an abnormal PCQ score were observed in patients in whom HRCT scan detected two or more lesions (+26.5%, p = 0.0045, McNemar test), asbestosis (+26.9%, p = 0.0067, McNemar test) and isolated pulmonary nodules (+21.5%, p = 0.0105, McNemar test). Conversely, 7.0% of subjects regained a normal PCQ score at T2 (data not shown).

**Table 2:** Comparison of the 3 subscales and global PCQ score at baseline and during follow-up according to descriptive variables

	Baseline	Follow-up	**P value**^**1**^
**PCQ Score**			
Social dimension score	2.67 (2.50-2.83)	3.36 (3.16-3.56)	P < 0.0001
Physical dimension score	2.61 (2.48-2.75)	3.23 (3.08-3.39)	P < 0.0001
Emotional dimension score	3.01 (2.87-3.15)	3.47 (3.32-3.62)	P < 0.0001
Global PCQ score	8.30 (7.91-8.69)	10.07 (9.62-10.53)	P < 0.0001
Abnormal PCQ score²	171 (20.6)	271 (32.6)	P < 0.0001
Acquiring abnormal PCQ score² at T2		158 (19.0)^3^	
	
**Gender**			
Males	8.24 (7.83-8.64)	10.11 (9.63-10.58)	P < 0.0001
Females	9.07 (7.67-10.47)	9.68 (8.03-11.33)	P = 0.4949
	P = 0.2601^4^	P = 0.6278^4^	
	
**Smoking status**			
Non-smokers	8.23 (7.65-8.80)	9.85 (9.18-10.50)	P < 0.0001
Former smokers	8.13 (7.55-8.71)	9.76 (9.08-10.43)	P < 0.0001
Smokers	9.52 (8.24-10.80)	12.75 (11.24-14.25)	P < 0.0001
	P = 0.1437^4^	P = 0.0013^4^	
	
**Counseling visit**			
Yes	8.03 (7.65-8.20)	9.98 (9.42-10.53)	P < 0.0001
No	8.88 (8.20-9.56)	10.29 (9.48-11.10)	P = 0.0002
	P = 0.0439^4^	P = 0.5244^4^	
	
**Self-assessment of asbestos exposure**			
Nil/light	6.14 (5.00-7.27)	7.44 (6.12-8.76)	P = 0.0074
Moderate	7.97 (7.37-8.57)	9.06 (8.35-9.76)	P = 0.0003
Heavy	9.96 (9.21-10.71)	12.43 (11.55-13.29)	P < 0.0001
Do not know	8.08 (7.28-10.27)	10.50 (9.56-11.53)	P < 0.0001
	P < 0.0001^4^	P < 0.0001^4^	

1: comparison of PCQ scores at baseline and during follow-up by t-test

2: PCQ values greater than the 95^th ^percentile of the PCQ score distribution in non-exposed subjects (see ref [16]

3: among the 661 subjects without an abnormal PCQ score at baseline

4: comparison of PCQ scores between variables at each time-point of the study by GLM

**Table 3:** Course of PCQ scores and proportions of subjects with abnormal PCQ score^a ^between T1 and T2 according to the HRCT scan results (n = 832, univariate analyses)

			At T1			At T2				Variation	
**CT scans**	**N**	**Abnormal PCQ**^**a **^**N (%)**	**Mean PCQ**	**P value**^**b**^	**Abnormal PCQ**^**a **^**N (%)**	**Mean PCQ**	**P value**^**b**^	**Acquiring** **Abnormal PCQ**^**a **^**N (%)**	**P value **^**c**^	**Mean PCQ**^**d**^	**P value**^**e**^

Normal	213	44 (20.7)	8.02	Reference	57 (26.8)	9.28	Reference	31 (14.6)	0.0633	+1.26	0.0010
Isolated pulmonary Nodules (only)	79	15 (19.0)	8.99	0.2019	27 (34.2)	10.58	0.1497	17 (21.5)	0.0105	+1.59	0.0276
Pleural plaques (only)	113	21 (18.6)	7.77	0.6972	40 (35.4)	11.24	0.0078	24 (21.2)	0.0004	+3.47	<0.0001
Asbestosis (with or without pleural plaques)	67	13 (19.4)	8.40	0.9280	26 (38.8)	10.64	0.1303	18 (26.9)	0.0067	+2.24	0.0005
Other diseases (only)	151	28 (18.5)	8.38	0.5490	53 (35.1)	10.19	0.2037	32 (21.2)	< 0.0001	+1.80	0.0002
Two or more of the above lesions	49	10 (20.4)	7.73	0.7504	21 (42.9)	10.86	0.1202	13 (26.5)	0.0045	+3.12	0.0002
Unknown results	160	40 (25.0)	8.79	0.2131	47 (29.4)	9.50	0.7556	23 (14.4)	0.2623	+0.71	0.1394

^a ^PCQ values greater than the 95^th ^percentile of the PCQ score distribution in non-exposed subjects (see ref [16])

^b ^Comparison of the PCQ score at baseline (t-test)

^c ^Comparison of the proportion of abnormal PCQ scores between T1 and T2 for each HRCT scan result (McNemar test)

^d ^ΔPCQ is defined as the PCQ at T2 - PCQ at T1 difference

^e ^Comparison between PCQ score at baseline and during follow-up for each HRCT scan result (paired t-test)

Follow-up of psychological impact showed a significant increase of the quantitative PCQ score (p < 0.0001, paired t-test). Baseline PCQ scores did not differ between HRCT scan groups. At T2, using the PCQ score of patients with normal CT scan as the reference, only patients with pleural plaques detected by CT scan showed a higher PCQ score (p = 0.0078, paired t-test). However, when comparing subjects to themselves between baseline and follow-up according to the results of HRCT scan, all subjects with a known result, even normal, demonstrated a significant increase of their PCQ score. The most marked increases of PCQ were observed for patients with isolated pleural plaques, followed by combinations of two or more lesions and abnormalities compatible with asbestosis. The presence of isolated pulmonary nodules was associated with a slight but significant increase of PCQ (8.99 to 10.58, p = 0.027, paired t-test) which was close to that observed in patients with normal HRCT scans (8.02 to 9.28, p = 0.001, paired t-test). Multivariate analyses taking gender, age, smoking status and self-assessment of asbestos exposure into account (Table 4) confirmed these findings. The increase of PCQ score was significant in all patients with a known result, and the most marked increase was observed for subjects with pleural plaques (+3.60 [95%CI: +2.15, +5.06], GLM). The presence of isolated pulmonary nodules was associated with a significant increase of the PCQ score of +1.88 [+0.34, +3.42], GLM). Even a normal HRCT scan result was associated with a significant increase (+1.40 [+0.11, +2.69], GLM). On pairwise comparisons, patients with pleural plaques demonstrated a significantly higher increase of PCQ score than that observed in patients with normal CT scan (p = 0.0042) and close to significance for patients with other disorders (p = 0.0597) or pulmonary nodules (p = 0.0734, data not shown). Finally, variables associated with the risk of developing an abnormal PCQ score according to our definition were tested by logistic regressions (Table 5). The presence of asbestosis and two or more diseases were associated with an almost significant risk (OR 1.92 [0.97-3.81] and OR 2.04[0.95-4.37], respectively), after adjustment for gender, age, smoking status, self-assessment of asbestos exposure and a final counseling visit.

**Table 4:** Main determinants of a significant variation of the PCQ score during follow-up (multivariate analysis, general linear model; n = 832)

Variables	**ΔPCQ**^**a,b **^**Mean increase**	95% CI	P value
*Gender*	-		0.1678
*Males*	+ 2.75	1.95, +3.54	
*Females*	+1.64	-0.05, +3.33	
*Smoking status*	-		0.0646
*Non smokers*	+1.71	+0.71, +2.71	
*Former Smokers*	+1.70	+0.57, +2.83	
*Smokers*	+3.17	+1.49, +4.85	
*Age (years)*	-		0.7027
*< 60*	+1.91	+0.91, +2.91	
*60-75*	+1.99	+0.95, +3.04	
*> 75*	+2.67	+0.78, +4.57	
*Self-assessment of asbestos exposure*	-		0.0095
Nil/light	+1.69	+0.11, +3.26	
Moderate	+1.38	+0.22, +2.55	
Heavy	+2.86	+1.64, +4.08	
Do not know	+2.84	+1.60, +4.07	
Counseling visit			0.2942
No	+1.96	+0.75, +3.16	
Yes	+2.43	+1.34, +3.52	
CT scan results	-		0.0039
Normal	+1.40	+0.11, +2.69	
Isolated pulmonary nodules (only)	+1.88	+0.34, +3.42	
Pleural plaques (only)	+3.60	+2.15, +5.06	
Asbestosis (with or without pleural plaques)	+2.52	+0.80, +4.24	
Other diseases (only)	+1.76	+0.46, +3.06	
Two or more of the above lesions	+3.37	+1.39, +5.34	
Unknown results	+0.83	-0.50, +2.17	

^a ^ΔPCQ is defined as the PCQ at T2-PCQ at T1 difference

^b ^Means are adjusted for gender, age, smoking status, counseling visit and self-assessment of asbestos exposure

**Table 5:** Main determinants of acquiring an abnormal PCQ score ^1 ^during follow-up (multivariate analysis, logistic regression model; n = 832)

Variables			
	OR	[95% CI]	P value
**Gender**			
**Males**	1	reference	
**Females**	0.88	[0.42-1.85]	0.7340
**Age**			
**< 60 years**	1	reference	
**60-74 years**	1.14	[0.76-1.70]	0.5125
**> = 75 years**	1.75	[0.83-3.54]	0.1675
**Smoking**			
**Non-Smoker**	1	reference	
**Former Smoker**	0.91	[0.62-1.36]	0.0577
**Smoker**	1.83	[1.00-3.34]	0.0242
**Counseling (yes)**			
**No**	1	reference	
**Yes**	1.35	[0.90-2.04]	0.1442
**Exposure**			
**Nil/light**	1	reference	
**Moderate**	1.24	[0.61-2.51]	0.0901
**Heavy**	2.34	[1.14-7.79]	0.0178
**Do not Know**	2.31	[1.12-4.77]	0.0275
**CT Scan Results**			
**Normal**	1	reference	
**Isolated pulmonary nodules (only)**	1.68	[0.86-3.31]	0.1313
**Pleural plaques (only)**	1.46	[0.79-2.69]	0.2315
**Asbestosis (with or without pleural plaques)**	1.92	[0.97-3.81]	0.0608
**Other diseases (only)**	1.49	[0.85-2.62]	0.1633
**Two or more of the above diseases**	2.04	[0.95-4.37]	0.0673
**Unknown results**	0.99	[0.53-1.83]	0.9775

^1^: Values of PCQ greater than the 95^th ^percentile of the PCQ score distribution in non-exposed subjects (see ref [16])

## Discussion

Initial HRCT scan screening in this previously asbestos-exposed population was associated with a significant increase of the PCQ score 6 months after the examination. This increase, relative to baseline values, concerned patients with an abnormal CT scan result, regardless of the abnormalities, but also normal CT scans, after adjustment for age, gender, smoking status self-assessment of asbestos exposure and final counseling visit. The most marked increase was observed in patients with pleural plaques that are benign lesions. In contrast, detection of isolated pulmonary nodules that may have potentially more serious consequences on health status was also associated with a less marked but significant increase of distress. However, only the presence of asbestosis or an association of two or more abnormalities appeared to be associated with a clinically significant modification of the PCQ score, although these associations were not statistically significant.

Numerous studies have focused on the impact of cancer screening on quality of life or distress at so-called "short-term" (usually < 3 months) or "long-term" (at 6 or 12 months depending on the studies). Not surprisingly, positive cancer screening can lead to anxiety [21]. Conversely, negative screening with a clear result is thought to be associated with only minimal and transient psychological impact [22-25]. It is well known that patients with lung cancer present a high rate of depression, with an average of 25% according to the review by Carlsen et al. [26]. However, to our knowledge, only two studies have evaluated the possible psychological consequences of lung cancer screening. Van den Berg [12] studied the effect of lung cancer screening in 351 subjects, at baseline, 1 day after the examination and 6 months later. Psychological impact was assessed using the Spielberger State-Trait Anxiety inventory (STAI-6), the 12-item Short Form (SF-12) and Impact of Event Scale (IES). No significant effect was seen between negative screening at baseline and negative repeated CT scan at 6 months. In the second study [11], 341 subjects were questioned four times (pre- and post-screening, 6 and 12 months) using the STAI and 3 questions of the PCQ. Patients with indeterminate results (namely one or more pulmonary nodule with advice to perform periodic survey) presented a significant increase in negative psychological measures immediately following screening, although these findings faded with time. No effect (positive or negative) was observed in subjects with negative screening. Vierikko et al. recently reported no significant negative psychological impact in subjects exposed to asbestos during the one-year survey of positive CT scan findings [13].

The results of our study differ from these findings, which are nevertheless difficult to interpret. Our study shows a significant increase of long-term distress as assessed by the PCQ score in patients in whom CT scan revealed an asbestos-related disease, and to a lesser extent, an isolated pulmonary nodule. Identical results have sometimes been reported for other types of cancer screening [25] but not for lung cancer. Several explanations can be proposed for these discrepancies. First, our study used a scale developed for a breast cancer screening program by mammography [19]. The choice to use this scale was primarily dictated by the aim of the study to assess the psychological consequences of radiological screening by HRCT scan in this population, and the existence of a validated French version of the scale. To our knowledge, no specific scale is available for the assessment of the psychological impact of CT scan screening for lung cancer or asbestos-related diseases. Recently published studies on the impact of CT scan screening for lung cancer used nonspecific quality-of-life scales. As stated by the authors themselves [11,12], these scales may be insensitive with regard to measurement of the specific impact of screening and some authors have recommended that these scales not be used for cancer screening [27]. Conversely, the authors of the PCQ considered that their tool can be used for a wide range of radiographic screening, as the PCQ scale explores three important dimensions of mental functions [19]. Our results support the hypothesis that most of the negative results concerning the psychological impact of screening may be due to the poor sensitivity of the scales used. One difficulty of the PCQ (as for other scales) is the absence of normal values for clinical interpretation [28]. Both univariate analyses using our definition of patients with an abnormal score or quantitative values of PCQ scores clearly showed positive associations between increasing distress and HRCT scan results, in contrast with published data. As an abnormal PCQ score was defined as a score greater than the 95th percentile in our reference group [16], we hypothesize that the psychological consequences associated with HRCT scan screening may affect quality of life in a sizeable proportion of subjects. Finally, the clinical significance of these results is difficult to determine, in view of the difference observed between multivariate GLM and LR models. Tested hypotheses and the statistical power, which clearly differed between the two statistical approaches, may easily explain these discordant results. As previously underlined, only one study has been conducted in an asbestos-exposed population with different results. However, this study used different tools to assess psychological consequences and a large proportion of the subjects of this study were surveyed over a long time, which may explain these discrepancies. Surprisingly, the presence of a counseling visit had no effect on psychological impact in our study [13]. Some authors have reported that distress may be modified by the perception of cancer risk for breast cancer screening [29,30] but also in lung cancer [31]. It can be hypothesized that the existence of asbestos exposure could lead to a greater fear of the consequences of screening in this particular population without any previous screening. The high level of distress observed at baseline in our study [16] supports this hypothesis as well as the increase associated with self-assessment of exposure. The slight but significant difference observed between subjects before attending a final visit also suggested a selection bias related to patients attending this visit. On the other hand, former smokers and especially current smokers enrolled in CT scan screening are thought to usually underestimate their risk of lung cancer [31,32]. Our findings obviously cannot be generalized to populations suitable for lung cancer screening, and further studies among ever smokers are needed to confirm our results. In particular, the role of perception of cancer risk in both asbestos-exposed subjects and ever smokers needs to be investigated.

Finally, a significant increase of PCQ score was also found in patients with normal HRCT scan after adjustment for gender, age, smoking status, counseling and self-assessment of asbestos exposure. These results have sometimes been reported in other screening studies for breast cancer [33] but not in lung cancer. Particular attention is required to explain these findings, if they are confirmed.

Several limitations of this study need to be discussed.

First of all, the increase in PCQ score observed in our population may not be clinically relevant, as no comparison with a control group of exposed subjects without HRCT was possible. In order to explore this limitation, we investigated subjects with no identified HRCT scan, but with a T2 PCQ questionnaire (after an interval of one year). Seventy-four patients were therefore retrieved and analyzed, showing no significant increase of their PCQ score (8.31 at T1 versus 9.20 at T2, p = 0.2452, data not shown). Analysis of our data using this group as reference instead of patients with normal HRCT scan results did not modify our main findings. Finally, no significant differences were observed between subjects for the baseline PCQ score before HRCT. As patients were compared to themselves at T2, observed differences were more likely to be related to HRCT results than to subject-related factors. However, a misclassification of this population cannot be excluded and these findings must be confirmed by another study using an appropriate control group.

Secondly, subjects volunteered to participate in the survey which could lead to a selection bias. In order to verify this hypothesis, we compared the PCQ score at baseline among respondents versus non-respondents to the survey, according to the presence or absence of a HRCT scan examination and found no statistical differences between these groups of subjects. Moreover, comparisons of PCQ score at baseline according to HRCT scan results showed no difference even with subjects who did not undergo CT scan (data not shown). We can therefore assume that, if it exists, a selection bias relative to psychological distress at baseline would have only a minimal effect on our results. On the other hand, HRCT scan results were not available for 160 patients. We reanalyzed our data assuming that all unknown HRCT scan results were normal. This analysis did not modify our results, particularly for the significant increase of PCQ score associated with normal HRCT scan results in multivariate analysis (data not shown).

A second point concerns the interval between HRCT scan examination and the second PCQ, fixed by the design at 6 months (as usually defined in the literature by "long-term"). This relatively long interval, albeit similar to that of other studies, may result in changes in health status, that may influence the second assessment of psychological impact, independently of the results of the initial HRCT scan. In order to minimize this possible bias, we reviewed our data after excluding patients with known cancer (lung cancers and mesotheliomas, 8 subjects) during follow-up. No changes in our results were observed. Variability in measurement of PCQ score also did not appear to be an explanation, as this score is considered to be reproducible [19,20]. However, anxiety scores may be affected by various other factors, even time, and a longer survey with more complete questionnaires, including general health questionnaires, would be useful. The development of a specific scale for asbestos-related health effects should be considered.

Finally, the definition used for HRCT scan results can also be questioned. For instance, the diagnosis of asbestosis is probably overestimated in these elderly subjects with nonspecific interstitial abnormalities. However, we decided to rate the presence of radiographic abnormalities on the basis of radiology reports, which allow more accurate assessment of the diagnosis given to the patients than a standardized and independent interpretation.

## Conclusion

This study reported that HRCT scan screening is associated with a long-term increase of distress in asbestos-exposed subjects. The most marked increase was observed for patients with pleural plaques that are considered to be a benign disease with no clinical consequences. According to previous results, this may suggest that asbestos workers have a specific and probably poorer perception of cancer risk than the general population, but these results must be confirmed in future studies in view of the difficulty of estimating the clinical significance of our findings. No clear guidelines have been established for screening for asbestos-related diseases, as no medical benefit has yet been demonstrated. Clear and specific information on CT scan screening and asbestos-related diseases must therefore be given to subjects before and after the examination in the context of individual screening. The possibility of psychological management must also be discussed with patients. A slight but significant increase of distress was also observed after normal HRCT scan results and detection of isolated pulmonary nodules in this population. These findings need to be studied further in subjects not exposed to asbestos. If confirmed, these results may have consequences for the CT scan screening for lung cancer, under the condition that current assessments of these programs provide any proof of the existence of a clear clinical benefit.

## Competing interests

The authors declare that they have no competing interests.

## Authors' contributions

CP and MM have made substantial contributions to conception and design of the study and interpretation of data and have been involved in drafting the Manuscript, AL and AS have made substantial contributions to analysis and interpretation of data; JCP and ML have been involved in revising the manuscript critically for important intellectual content; All authors have given final approval of the version to be published.

## Pre-publication history

The pre-publication history for this paper can be accessed here:

http://www.biomedcentral.com/1471-2458/10/647/prepub

## Supplementary Material

Additional file 1 **PCQ questionnaire**. This file provides the PCQ questionnaire as used in this study, adapted from Cockburn et al. Note that the questions and the 3 axis have not been modified Click here for file

## References

[B1] Diagnosis and initial management of nonmalignant diseases related to asbestos Am J Respir Crit Care Med 2004 170 6 691 715 10.1164/rccm.200310-1436ST 15355871

[B2] Gustavsson P Nyberg F Pershagen G Scheele P Jakobsson R Plato N Low-dose exposure to asbestos and lung cancer: dose-response relations and interaction with smoking in a population-based case-referent study in Stockholm, Sweden Am J Epidemiol 2002 155 11 1016 1022 10.1093/aje/155.11.1016 12034580

[B3] Paris C Benichou J Raffaelli C Genevois A Fournier L Menard G Broessel N Ameille J Brochard P Gillon JC Gislard A Letourneux M Factors associated with early-stage pulmonary fibrosis as determined by high-resolution computed tomography among persons occupationally exposed to asbestos Scand J Work Environ Health 2004 30 3 206 214 15250649 10.5271/sjweh.781

[B4] Clin B Morlais F Guittet L Gislard A Marquignon M Paris C Caillard J Launoy G Letourneux M Performance of chest radiograph and CT scan for lung cancer screening in asbestos-exposed workers Occup Environ Med 2009 66 8 529 34 10.1136/oem.2008.041525 19273475

[B5] Black WC Computed tomography screening for lung cancer: review of screening principles and update on current status Cancer 2007 110 11 2370 2384 10.1002/cncr.23059 17941031

[B6] Tossavainen A International expert meeting on new advances in the radiology and screening of asbestos-related diseases Scand J Work Environ Health 2000 26 5 449 454 11103845

[B7] Das M Muhlenbruch G Mahnken AH Hering KG Sirbu H Zschiesche W Knoll L Felten MK Kraus T Gunther RW Wildberger JE Asbestos Surveillance Program Aachen (ASPA): initial results from baseline screening for lung cancer in asbestos-exposed high-risk individuals using low-dose multidetector-row CT Eur Radiol 2007 17 5 1193 1199 10.1007/s00330-006-0426-8 17047960

[B8] Fasola G Belvedere O Aita M Zanin T Follador A Cassetti P Meduri S De Pangher V Pignata G Rosolen V Barbone F Grossi F Low-dose computed tomography screening for lung cancer and pleural mesothelioma in an asbestos-exposed population: baseline results of a prospective, nonrandomized feasibility trial--an Alpe-adria Thoracic Oncology Multidisciplinary Group Study (ATOM 002) Oncologist 2007 12 10 1215 1224 10.1634/theoncologist.12-10-1215 17962615

[B9] Mulshine JL Sullivan DC Clinical practice. Lung cancer screening N Engl J Med 2005 352 26 2714 2720 10.1056/NEJMcp042630 15987920

[B10] Schnoll RA Bradley P Miller SM Unger M Babb J Cornfeld M Psychological issues related to the use of spiral CT for lung cancer early detection Lung Cancer 2003 39 3 315 325 10.1016/S0169-5002(02)00501-9 12609570

[B11] Byrne MM Weissfeld J Roberts MS Anxiety, fear of cancer, and perceived risk of cancer following lung cancer screening Med Decis Making 2008 28 6 917 925 10.1177/0272989X08322013 18725404

[B12] van den Bergh KA Essink-Bot ML Bunge EM Scholten ET Prokop M van Iersel CA van Klaveren RJ de Koning HJ Impact of computed tomography screening for lung cancer on participants in a randomized controlled trial (NELSON trial) Cancer 2008 113 2 396 404 10.1002/cncr.23590 18484588

[B13] Vierikko T Kivisto S Jarvenpaa R Uitti J Oksa P Virtema P Vehmas T Psychological impact of computed tomography screening for lung cancer and occupational pulmonary disease among asbestos-exposed workers Eur J Cancer Prev 2009 18 3 203 206 10.1097/CEJ.0b013e328329d800 19728402

[B14] Meyerowitz BE Sullivan CD Premeau CL Reactions of asbestos-exposed workers to notification and screening Am J Ind Med 1989 15 4 463 475 10.1002/ajim.4700150410 2729288

[B15] Lowinger P Asbestos and other toxins Am J Public Health 1990 80 10 1274 10.2105/AJPH.80.10.1274 2400048 PMC1404818

[B16] Maurel M Stoufflet A Thorel L Berna V Gislard A Letourneux M Pairon JC Paris C Factors associated with cancer distress in the Asbestos Post-Exposure Survey (APEXS) Am J Ind Med 2009 52 4 288 296 10.1002/ajim.20672 19152347

[B17] Paris C Thierry S Brochard P Letourneux M Schorle E Stoufflet A Ameille J Conso F Pairon JC Pleural plaques and asbestosis: dose and time-response relationships based on HRCT data Eur Respir J 2009 34 1 72 9 10.1183/09031936.00094008 19129281

[B18] MacMahon H Austin JH Gamsu G Herold CJ Jett JR Naidich DP Patz EF Jr Swensen SJ Guidelines for management of small pulmonary nodules detected on CT scans: a statement from the Fleischner Society Radiology 2005 237 2 395 400 10.1148/radiol.2372041887 16244247

[B19] Cockburn J De Luise T Hurley S Clover K Development and validation of the PCQ: a questionnaire to measure the psychological consequences of screening mammography Soc Sci Med 1992 34 10 1129 1134 10.1016/0277-9536(92)90286-Y 1641674

[B20] Maziade J Thomassin L Morin R [Emotional, physical and social consequences of breast cancer: viability and utilization of a clinical questionnaire] Can J Public Health 2001 92 1 57 61 11257994 10.1007/BF03404846 PMC6980022

[B21] Lerman C Miller SM Scarborough R Hanjani P Nolte S Smith D Adverse psychologic consequences of positive cytologic cervical screening Am J Obstet Gynecol 1991 165 3 658 662 1892194 10.1016/0002-9378(91)90304-a

[B22] Brett J Bankhead C Henderson B Watson E Austoker J The psychological impact of mammographic screening. A systematic review Psychooncology 2005 14 11 917 938 10.1002/pon.904 15786514

[B23] Sutton S Saidi G Bickler G Hunter J Does routine screening for breast cancer raise anxiety? Results from a three wave prospective study in England J Epidemiol Community Health 1995 49 4 413 418 10.1136/jech.49.4.413 7650466 PMC1060131

[B24] Ekeberg O Skjauff H Karesen R Screening for breast cancer is associated with a low degree of psychological distress Breast 2001 10 1 20 24 10.1054/brst.2000.0177 14965553

[B25] Cullen J Schwartz MD Lawrence WF Selby JV Mandelblatt JS Short-term impact of cancer prevention and screening activities on quality of life J Clin Oncol 2004 22 5 943 952 10.1200/JCO.2004.05.191 14990651

[B26] Carlsen K Jensen AB Jacobsen E Krasnik M Johansen C Psychosocial aspects of lung cancer Lung Cancer 2005 47 3 293 300 10.1016/j.lungcan.2004.08.002 15713512

[B27] Brodersen J Thorsen H Cockburn J The adequacy of measurement of short and long-term consequences of false-positive screening mammography J Med Screen 2004 11 1 39 44 10.1258/096914104772950745 15006113

[B28] Green JM Hewison J Bekker HL Bryant LD Cuckle HS Psychosocial aspects of genetic screening of pregnant women and newborns: a systematic review Health Technol Assess 2004 8 33 1 109 iii, ix-x 10.3310/hta8330 15298822

[B29] van Dooren S Rijnsburger AJ Seynaeve C Duivenvoorden HJ Essink-Bot ML Tilanus-Linthorst MM de Koning HJ Tibben A Psychological distress in women at increased risk for breast cancer: the role of risk perception Eur J Cancer 2004 40 14 2056 2063 10.1016/j.ejca.2004.05.004 15341979

[B30] Henderson BJ Tyndel S Brain K Clements A Bankhead C Austoker J Watson E Duffy S Evans G Fielder H Gray J Mackay J Macmillan D Factors associated with breast cancer-specific distress in younger women participating in a family history mammography screening programme Psychooncology 2008 17 1 74 82 10.1002/pon.1201 17410528

[B31] Bunge EM van den Bergh KA Essink-Bot ML van Klaveren RJ de Koning HJ High affective risk perception is associated with more lung cancer-specific distress in CT screening for lung cancer Lung Cancer 2008 62 3 385 390 10.1016/j.lungcan.2008.03.029 18468717

[B32] Hahn EJ Rayens MK Hopenhayn C Christian WJ Perceived risk and interest in screening for lung cancer among current and former smokers Res Nurs Health 2006 29 4 359 370 10.1002/nur.20132 16847914

[B33] Swanson V McIntosh IB Power KG Dobson H The psychological effects of breast screening in terms of patients' perceived health anxieties Br J Clin Pract 1996 50 3 129 135 8733330

