# Supplementary material for: CT scan screening is associated with increased distress among subjects of the APExS
Source: BMC Public Health. 2010 Oct 26;10:647. doi: 10.1186/1471-2458-10-647 (PMC2988732; doi:10.1186/1471-2458-10-647)
Supplement: Additional file 1 — PCQ questionnaire. This file provides the PCQ questionnaire as used in this study, adapted from Cockburn et al. Note that the questions and the 3 axis have not been modified [file 1471-2458-10-647-S1.DOC]

**Additional file 1: PCQ QUESTIONNAIRE (adapted from Cockburn, 1992)**

We would like to find about the subject’s experience of the Asbestos Post-Exposure Survey. Would you therefore please answer the questions on this questionnaire as best you can.

*Over the last week* how often have you experienced the following things because of *thoughts and feelings about asbestos-related diseases:*

| scale | item | Not at all | Rarely | Some of the time | Quite a lot of the time |
| --- | --- | --- | --- | --- | --- |
| P | Had trouble sleeping | 0 | 1 | 2 | 3 |
| P | Experienced a change in appetite | 0 | 1 | 2 | 3 |
| E | Been unhappy or depressed | 0 | 1 | 2 | 3 |
| E | Been scared and panicky | 0 | 1 | 2 | 3 |
| E | Felt nervous or strung up | 0 | 1 | 2 | 3 |
| E | Felt under strain | 0 | 1 | 2 | 3 |
| S | Found you have been keeping things from those who are close to you | 0 | 1 | 2 | 3 |
| S | Found yourself taking things out on other people | 0 | 1 | 2 | 3 |
| S | Found yourself noticeably withdrawing from those who are close to you | 0 | 1 | 2 | 3 |
| P | Had difficulty doing things around the house which you normally do | 0 | 1 | 2 | 3 |
| P | Had difficulty meeting work and other commitments | 0 | 1 | 2 | 3 |
| E | Felt worried about your future | 0 | 1 | 2 | 3 |

P =physical axis; e= emotional axis; s = social axis.
